# Explainable AI framework for improved Thalassemia mental health classification and feature selection

**DOI:** 10.1371/journal.pone.0341168

**Published:** 2026-01-23

**Authors:** Shahriar Siddique Ayon, Abdullah Al Mamun, Md. Ebrahim Hossain, Wasan Alamro, Yazan M. Allawi, Nuzhat Noor Islam Prova, Md. Saef Ullah Miah, Salman Md Sultan, Ahmad Abadleh

**Affiliations:** 1 Department of Computer Science and Engineering, American International University-Bangladesh (AIUB), Dhaka, Bangladesh; 2 Department of Computer Science and Engineering, Dhaka University of Engineering and Technology (DUET), Gazipur, Bangladesh; 3 Department of Communications and Computer Engineering, Faculty of Engineering, Al-Ahliyya Amman University, Amman, Jordan; 4 Department of Electrical Engineering, College of Engineering, Princess Nourah bint Abdulrahman University, Riyadh, Saudi Arabia; 5 Department of Computer Science and Information Systems, Pace University, Seidenberg School, New York, New York, United States of America; 6 Innovative Skills LTD, Dhaka, Bangladesh; 7 CS Department, Mutah University, Jordan; The University of Jordan, JORDAN

## Abstract

Mental health challenges in Thalassemia patients are often overlooked, despite their significant impact on quality of life. Traditional statistical and machine learning approaches often fail to capture the complex, nonlinear relationships between psychosocial and clinical variables, limiting both accuracy and interpretability. To overcome this, we propose the Adaptive Multi-Stage Ensemble with Dynamic Feature Interaction (AMSE-DFI)—a novel feature selection framework that dynamically integrates mutual information, ensemble learning, and graph attention mechanisms to capture intricate feature interdependencies often missed by traditional approaches. Using the SF-36 health survey data from 356 Bangladeshi patients, AMSE-DFI effectively identified key predictors such as total SF score, role emotional, and physical health summary, which collectively reflect both physical and psychological well-being. The model outperformed conventional approaches, showing strong predictive reliability and robust generalization, with SMOTE effectively addressing class imbalance in the clinical data. Importantly, Local Interpretable Model-Agnostic Explanations (LIME) based explainable AI offered clear, interpretable insights into how key features affect individual patient outcomes, making the model more understandable and actionable for clinicians. This framework provides a practical, transparent tool to support early detection and personalized management of mental health challenges in Thalassemia care.

## 1 Introduction

Thalassemia is a common inherited blood disorder caused by abnormal hemoglobin production. Due to its chronic nature, permanent physical changes, and lifelong treatment requirements, pediatric patients with Thalassemia major are more prone to developing mental disorders and cognitive impairments [1]. The global burden of disease Survey estimates that approximately 1.31 million people live with severe Thalassemia worldwide, affecting 358 million carriers and causing around 11,100 deaths annually [2]. More than 300,000 babies are born with the hereditary blood disorder Thalassemia each year worldwide and it is most common in South and Southeast Asia, the Middle East, and the Mediterranean region [3–5].

Thalassemia affects not only physical health but also mental and emotional well-being. Patients often experience chronic fatigue, social stigma, and a strong dependence on healthcare services. Beyond the physiological burden, thalassemia has a profound psychosocial impact on patients and their families [6]. Mental health disorders, including depression and anxiety, are significantly more prevalent among Thalassemia patients than in the general population, negatively affecting treatment adherence, illness perception, and overall quality of life [7,8]. Despite their clinical importance, mental health issues in Thalassemia patients remain underexplored, with conventional assessments relying on self-reports or clinician scales that are prone to bias and cultural variability [9]. Furthermore, the multifactorial nature of psychological disorders—shaped by demographic, clinical, biochemical, and social determinants—renders manual assessment methods inconsistent and often inadequate. These limitations underscore the pressing need for automated, data-driven frameworks capable of facilitating early and accurate detection of mental health conditions in Thalassemia patients [10,11].

Recent advances in Artificial Intelligence (AI) and Machine Learning (ML) have demonstrated substantial potential in medical data analysis and predictive modeling. These technologies excel at uncovering complex, nonlinear relationships among diverse clinical and behavioral variables, enabling the early detection of disease patterns that may elude conventional diagnostic approaches. In the domain of mental health, ML-based classification systems have been successfully deployed for tasks such as depression detection, anxiety prediction, and suicide risk assessment, offering scalable and data-driven alternatives to traditional screening methods [12]. A recent study of adults with Thalassemia found that over one-third experienced anxiety and depression, with many also showing poor sleep and fatigue, strongly linked to ferritin and hemoglobin levels [13]. Mediani et al. [14] observed that 70.5% of adolescents exhibited mild to severe anxiety, with self-esteem and coping mechanisms identified as key factors. Pan, j. et al. [15] report that the prevalence of anxiety and depression among Thalassemia carriers receiving genetic counseling in Taizhou was 3.7% for each condition. Zafar, M. et al. [16] applied the Chi-square test to examine the association between sociodemographic characteristics and the prevalence of mental disorders. The study revealed that among the participants 16% experienced severe anxiety 20% suffered from depression 5% were diagnosed with psychotic disorders. The use of these techniques in chronic diseases like Thalassemia is still limited, presenting an opportunity to apply ML-driven mental health analytics for early intervention and better patient outcomes.

To overcome these challenges, some previous research has mainly relied on statistically based traditional ML methods, such as regression analysis, ensemble methods (e.g, XGBoost) which struggle to capture the complex nature of mental health [17–19]. As a result, subtle predictors of psychological distress are often overlooked, particularly when analyzing multidimensional data such as patient-reported outcomes from instruments like the SF-36 questionnaire [20]. A key challenge in adopting AI in healthcare is the lack of transparency in “black-box” models, which, despite high accuracy, cannot explain their decisions. This has driven the rise of Explainable AI (XAI), aimed at making AI predictions transparent, understandable, and clinically actionable. XAI offers a powerful solution by enabling interpretability and transparency in predictive modeling [21].

In this study, we propose an XAI-driven framework for predicting psychological distress in patients with Thalassemia by integrating SF-36 health survey responses with key demographic and hematological features, enabling a multidimensional assessment of mental health. To identify the most influential predictors, we employ advanced feature selection techniques, including Recursive Feature Elimination (RFE), mutual information, chi-squared analysis, and ANOVA F-tests, while addressing class imbalance with SMOTE to improve model generalization and stability. The framework evaluates ten leading ML and deep learning (DL) models, applying dimensionality reduction and our Adaptive Multi-Stage Ensemble with Dynamic Feature Interaction (AMSE-DFI) method for refined feature selection. Among these, the PSO-based Light Gradient Boosting Machine (LGBM) achieved the highest accuracy. Finally, Local Interpretable Model-Agnostic Explanations (LIME) provide interpretable insights into both positive and negative feature contributions, offering a transparent, data-driven approach to support clinicians and researchers in predicting psychological distress and guiding informed mental health interventions. The key contributions of this paper are:

- Develop an AMSE-DFI feature selection method that enhances the predictive accuracy for mental health outcomes in Thalassemia patients.
- Analyze and compare target column-based features to generate new insights into mental health indicators.
- Utilize XAI’s LIME to provide clear insights into how key features influence predictions and contribute to increasing model transparency.
- Validate the proposed framework on a Thalassemia dataset, demonstrating superior performance compared to existing methods.

The rest of the paper is organized as follows: Sect 2 provides an overview of recent studies on feature selection and ML for mental health prediction. Sect 3 details the dataset, preprocessing procedures, and techniques used in this investigation. Sect 4 presents the XAI analysis and experimental findings. Sect 5 offers a comprehensive explanation of the results, emphasizing the model’s interpretability and usefulness. Finally, Sect 6 summarizes our findings presented in this paper and offers suggestions for future research.

## 2 Related works

Thalassemia is a chronic hereditary condition that requires lifelong medical management, leading to various psychosocial and physiological challenges. Patients often experience depression, anxiety, and a diminished quality of life due to continuous blood transfusions, iron chelation therapy, and the social stigma associated with the disease [22]. Anxiety and depressive symptoms are especially prevalent among adolescents and young adults, largely due to treatment burdens and disease complications [23]. Most epidemiological studies are cross-sectional, rely on self-report instruments, and rarely integrate objective hematological markers with patient-reported outcomes for predictive modeling.

The concept of mental health is complex and includes social, psychological, and emotional well-being, all of which have an impact on people’s thoughts, feelings, and behaviors. Mushtaq et al. [24] conducted a study on 100 Thalassemia patients (aged 12–18 years) and found that pain perception was significantly associated with pain-related anxiety, negative affect, and depression. Similarly, Vadakkiniath et al. [25] reported high prevalence rates of stress (68.7%), anxiety (51.1%), and depression (58.8%) among chronic illness patients, highlighting the impact of mental disorders on treatment adherence and quality of life. Sharma et al. [26] emphasized that stigma and insufficient diagnostic resources continue to hinder effective mental health care. These studies generally target genetic or hematologic screening rather than psychosocial or mental health outcomes, and they often lack interpretability analyses linking physiological indicators to psychological status.

The psychological burden of Thalassemia is further reflected in several population-based studies. Alhaj et al. [27] identified high prevalence of depression (72%), anxiety (79%), and stress (69%) symptoms among Thalassemia patients. Ayon et al. [28] observed severe anxiety predominantly among males aged 18–26 and females aged 18–22. Additionally, Hiradfar and Noorazar [29] found that nearly 80% of children with Thalassemia major or intermediate exhibited psychological disorders, with oppositional defiant disorder (ODD) and social phobia being most common. Neurocognitive impairments in transfusion-dependent Thalassemia (TDT) have been associated with subclinical brain hemosiderosis, as shown by MRI findings correlating with lower IQ and heightened anxiety [30].

AI and ML have increasingly been used to enhance diagnostic precision and disease management in Thalassemia. Humayun et al. [31] developed a hybrid model combining logistic regression and neural networks, achieving 97.81% prediction accuracy for mental health outcomes in Thalassemia patients. Fu et al. [32] implemented an SVM model using data from 350 anemic adults, achieving an average AUC of 0.76 and outperforming traditional indices. Similarly, Basu et al. [33] applied Random Forest, XGBoost, and Decision Tree algorithms to stratify TDT patients, with their Multi-Dimensional Assessment (MDA) model achieving *R*^2^ = 0.947 and RMSE=1.247. Al-Hakeim et al. [34] further demonstrated the influence of iron overload and inflammation on depressive symptom subdomains.

Beyond traditional ML, AI-driven frameworks have significantly improved Thalassemia detection accuracy. For instance, [35] reported that XGBoost achieved 90.11% accuracy in detecting *α*-thalassemia. Wiratchawa et al. [36] demonstrated a DL-based model with 86% accuracy in classifying *β*-thalassemia subtypes, while Zhang et al. [37] utilized AI techniques to identify Thalassemia carriers from blood morphology images with high reliability. Vaishnavi et al. [38] found the Stacking method to outperform other ML models (accuracy = 81.75%). Many models report strong in-sample performance but rely on limited sample sizes, lack rigorous external validation, and do not consistently address class imbalance or feature redundancy.

Feature selection has proven vital for improving ML-based mental health classification in Thalassemia. Traditional methods—such as chi-squared, ANOVA, mutual information (MI), and recursive feature elimination (RFE)—enhance model performance by eliminating irrelevant features. Saleem et al. [39] showed that combining Chi-Square feature selection with Linear Regression achieved 91.56% accuracy, 91.04% precision, and 92.65% recall. Nadimpalli et al. [40] identified the lack of optimized feature selection strategies as a major gap in mental health monitoring. The inability of traditional methods to manage complex, non-linear datasets compromises prediction reliability, as observed by Pratham et al. [41], where Decision Tree models achieved the highest accuracy (83.05%) among other tested algorithms. Azijah et al. [42] further advanced this domain by integrating ELM, MLP, and CatBoost, achieving 92.76% accuracy in mental health classification. Existing feature selection techniques applied in Thalassemia and mental health settings rarely combine interaction-aware selection with explainability constraints, which can limit clinical interpretability.

Recently, the integration of XAI techniques with ML models has enhanced both predictive accuracy and model transparency. Ko et al. [43] achieved 93.43% classification accuracy using wearable-based DL models supported by SHAP and LIME for interpretability. Magboo et al. [44] used LIME to enhance the transparency of logistic regression models for depression prediction, reaching 91% accuracy. Similarly, Bashar et al. [45] applied a hybrid clustering-classification approach using Random Forest with 97.93% accuracy, identifying PTSD and depression symptoms as critical predictors. The increasing adoption of XAI methods in mental health assessments demonstrates their effectiveness in revealing feature importance and decision logic [46,47]. Moreover, DL-based diagnostic models have revolutionized Thalassemia screening, particularly for image-based tasks such as hemoglobin electrophoresis analysis [48]. Combining ML with XAI thus provides a pathway toward reliable, interpretable, and clinically applicable models for patient care. Optimization approaches may overfit if cross-validation and resampling procedures are not applied correctly, and the interpretability of optimized ensembles can suffer without explicit XAI mechanisms.

Overall, the reviewed studies underscore the dual burden of Thalassemia—physiological and psychological—and the growing potential of ML and XAI in improving mental health classification, diagnostic precision, and interpretability. Few studies combine hematological markers, SF-36 patient-reported outcomes, and demographic/social features to predict psychological outcomes in Thalassemia cohorts. Many high-accuracy claims lack external benchmarking, class-imbalance handling, or local explainability necessary for clinical decision-making. These gaps motivate the development of our AMSE-DFI + XAI framework, which integrates optimized feature selection with explainable ML to provide reliable, interpretable, and clinically relevant mental health predictions. Table 1 summarizes the key contributions, sample sizes, methodologies, and limitations of the studies discussed.

**Table 1:** Summary of related works.

Study	Sample size	Method	Result	Key Limitation
[24]	100 patients	Cross-sectional design	The study’s findings include pain anxiety, negative affect, depression, and anxiety disorders	Does not integrate predictive modeling.
[49]	486 patients	Statistical analysis	AUC of 0.981	The study relied solely on traditional statistical analyses.
[31]	310 samples	MLR, SHAP, LIME	Accuracy of 97.81%	Class imbalance was present (19% positive cases), and class-balancing techniques such as SMOTE or cost-sensitive learning were not employed,
[27]	160 patients	Cross-sectional questionnaire-based	Anxiety, stress, and depression scores were 2.41, 1.68, and 1.80	The study did not employ machine learning or advanced predictive modeling techniques.
[32]	350 patients	Support Vector Machine	AUC of 0.76	SVM outperformed traditional indices but achieved only moderate accuracy.
[34]	111 samples	Machine learning	Identified depressive symptom subdomains and their relationship with iron overload and inflammatory biomarkers	No mention of external validation or independent testing; used only cluster analysis and partial least squares.
[33]	105 samples	MDA, XGBoost, RF, Decision Tree	*R*^2^ = 0.947, RMSE = 1.247	High accuracy achieved by XGBoost (100%) without external validation suggests overfitting risk.

In summary, while significant progress has been made in ML-based Thalassemia screening and mental health prediction, few studies have bridged these domains. Traditional methods often fail to capture the complex links between psychosocial and physiological factors, leading to less accurate mental health classification. This study addresses that gap by introducing an interpretable ML–XAI framework that combines optimized feature selection with XAI, improving prediction accuracy and supporting personalized mental health care for Thalassemia patients.

## 3 Methodology

**Fig 1:**
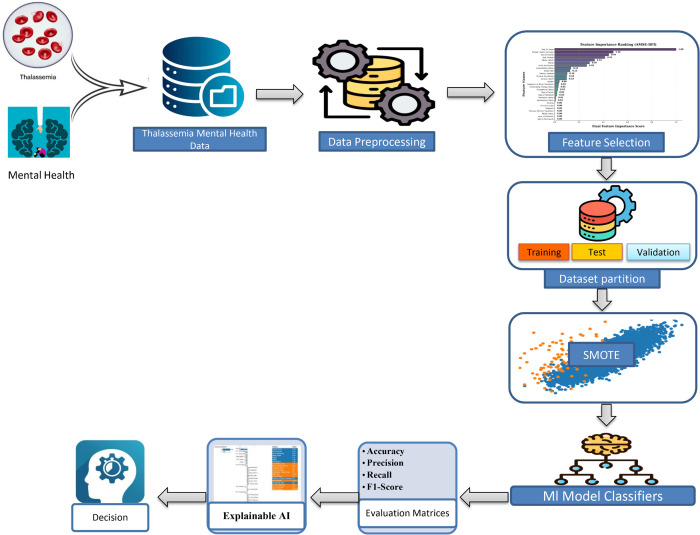
Proposed methodology for mental health classification in Thalassemia patients.

This study follows a structured approach, starting with data collection and preprocessing, followed by feature selection, ML model selection, hyperparameter tuning, and oversampling. Finally, an XAI framework is used to evaluate model predictions. Fig 1 visually summarizes the entire methodology, providing a clear and structured overview of our approach.

### 3.1 Data collection and description

We used a Mendeley dataset that records health-related quality of life metrics from Bangladeshi Thalassemia patients measured by the SF-36 questionnaire [20]. Furthermore, some key findings reported these data have been published in a reported journal [50], which we have reviewed in the literature section.

This dataset includes a rich set of categorical variables capturing participants’ demographic, socioeconomic, and clinical characteristics. In terms of gender, respondents were classified as either Male or Female, while marital status was recorded as Single or Married. Family structure was categorized into Nuclear, Joint, and Broken families. Educational attainment ranged from Illiterate to Master of Science, encompassing Primary school, Secondary school, High school, Bachelor of Science, and can read and write. Participants’ occupational status included High school student, University student, Civil servant, Self-employed, Housewife, and Unemployed. Residence was divided into Urban and Rural areas, and economic background was classified as Low income, Moderate income, or High income. On the clinical side, the type of Thalassemia diagnosis comprised *β*-Thalassemia major, E-*β*-Thalassemia, Hemoglobin H, and Others, with disease severity labeled as Yes or No. Transfusion dependency was recorded as either Transfusion dependent or Transfusion independent, while transfusion frequency was categorized as High (13–22 times/year), Low (6–12 times/year), or Occasional (1–5 times/year). Additional clinical details included whether participants had received a transfusion in the previous three months (Yes or No), had undergone splenectomy (Yes or No), or were affected by comorbidities such as Hepatitis B or C, Diabetes mellitus, Cardiological disorders, or Others. Finally, compliance with iron chelating therapy was categorized as Good compliance or Poor compliance.

In addition, our data set comprises medical expenses and overall health assessments, which includes general health, physical abilities, pain levels, energy levels, emotional well-being, and social interactions. Finally, it provides summary scores for physical and mental health, offering a comprehensive view of how Thalassemia affects daily life. The dataset focuses on the mental and physical health of 356 Thalassemia patients, encompassing 30 features, with 54.21% males and 45.79% females. The target variable classifies patients into two groups: ‘good’ and ‘bad’ health, providing valuable insights to understand the broader impact of Thalassemia. The data set contains both categorical and numerical features, with Table 2 displaying the mean, standard deviation (std. dev), and the value ranges (minimum and maximum) for the numerical features.

**Table 2:** Detailed description of the dataset’s numerical features.

No.	Feature	Mean	Std. Dev.	Range (Min–Max)
1	Age of Participants	19.75	8.02	11–53
2	Medical Expense	84,410.11	70,446.08	500–420,000
3	General Health	48.19	16.94	0–85
4	Physical Functioning	72.47	27.01	0–100
5	Role Physical	51.05	37.77	0–100
6	Bodily Pain	71.52	27.57	0–100
7	Vitality	59.44	19.76	0–100
8	Mental Health	63.47	18.81	4–100
9	Role Emotional	58.52	38.59	0–100
10	Social Functioning	44.24	23.33	0–100
11	Physical Health Summary	64.55	18.05	0–98.3
12	Total SF Score	61.24	16.68	0–93.1

### 3.2 Data preprocessing

**Fig 2:**
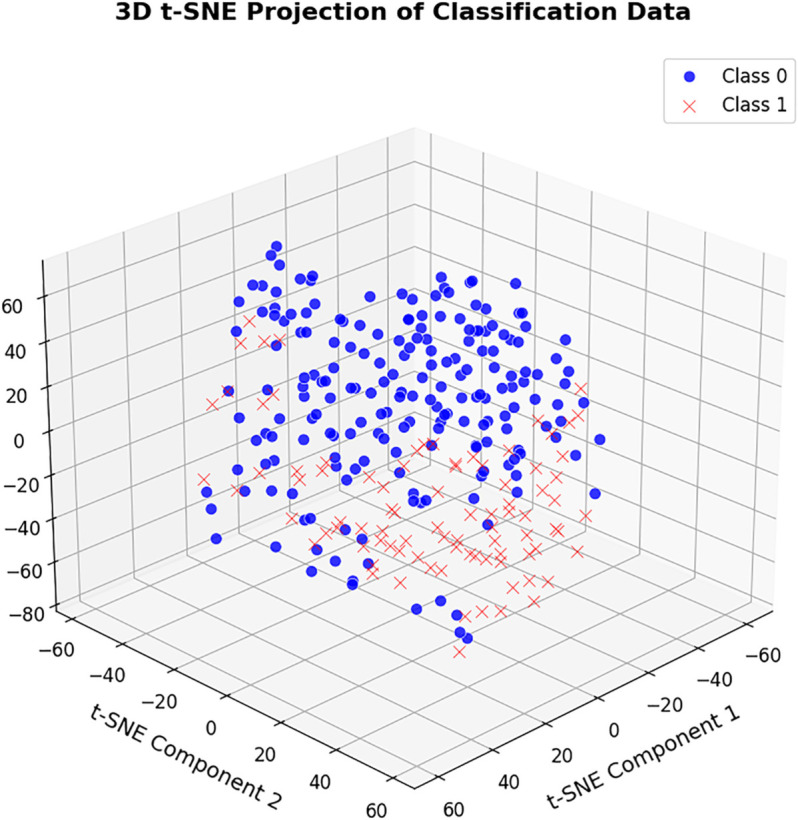
3D t-SNE visualization of classification data: class 0 ("Good") vs. class 1 ("Bad").

After collecting the data, we preprocessed the dataset focusing on the health conditions of Thalassemia patients. Subsequently, there was no missing values identified and the unnecessary features that are unrelated to our study were removed. All categorical variables, including the target column, were converted into numerical form using label encoding. Without scaling, the numerical features contained many outliers, which negatively affected model performance [51].To address this, all numerical variables were standardized, adjusting them to a uniform range before model training. The final dataset consists of 28 features and 356 data points. The target column, Mental_Health_Status, consists of 245 “Good” and 111 “Bad” health status records. To show the data’s structure, we used t-SNE (t-Distributed Stochastic Neighbor Embedding), a method for reducing high-dimensional data to 2D or 3D. Fig 2 shows a 3D t-SNE plot of the data, with Class 0 ("Good") and Class 1 ("Bad") displayed across three t-SNE components. The plot shows clusters reflecting patient groups with similar mental health outcomes, indicating that the selected features effectively separate classes and highlight the model’s ability to distinguish mental health status. After testing multiple data split ratios, we selected an 80:20 train-test split with 20% of the training data used for validation, which provided the best accuracy without overfitting. This resulted in 228 training samples, 71 testing samples, and 57 validation samples from the training set, out of 356 total data points. A total of 57 data points were reserved for validation, selected based on ratio-based splitting to optimize model performance and avoid overfitting.

### 3.3 Feature selection

Choosing the right features is key to improving ML models. It helps the model run faster, avoids overfitting, and makes predictions more accurate. Using too few features can limit the model’s ability, while too many can slow it down and reduce effectiveness. This study explores different ways to select the most important features for better results. In this study, we explored six feature selection methods, including our proposed AMSE-DFI. These methods fall into three categories: filter-based (Chi-Squared, ANOVA, Mutual Information), wrapper-based (RFE), and embedded-based (RFE with MI). Additionally, our AMSE-DFI method combines different approaches for better feature selection. For experimental purposes, we removed the features with the lowest scores and implemented all techniques using scikit-learn. Table 3 presents the selected features for the dataset.

**Table 3:** Feature selection methods and selected features for the dataset.

Main Features	Chi-Squared	ANOVA	MI	RFE	RFE-MI	AMSE-DFI
Age of Participants	✓	✓	×	✓	✓	×
Gender	✓	✓	✓	✓	✓	✓
Marital Status	✓	✓	✓	×	×	×
Type of Family	✓	✓	×	×	✓	✓
Level of Education	×	×	✓	✓	✓	×
Occupational Status	×	×	✓	✓	×	✓
Area of Residence	✓	×	×	×	×	✓
Economic Class	×	×	✓	✓	✓	×
Diagnosis	×	×	×	×	✓	×
Severity	×	×	×	×	✓	×
Transfusion Status	✓	×	×	×	×	✓
Frequency of Blood Transfusion	×	×	✓	✓	×	✓
Previous 3 Months Transfusion	×	×	×	×	×	×
Splenectomy Status	✓	×	×	×	×	✓
Comorbidities Status	×	×	✓	✓	✓	✓
Ironchelating Therapy Status	×	×	✓	×	×	✓
Medical Expense	×	×	✓	✓	✓	✓
General Health	✓	✓	✓	✓	✓	✓
Physical Functioning	✓	✓	✓	✓	✓	✓
Role Physical	✓	✓	✓	✓	✓	✓
Bodily Pain	✓	✓	✓	✓	✓	✓
Vitality	✓	✓	✓	✓	✓	✓
Mental Health	✓	✓	✓	✓	✓	✓
Role Emotional	✓	✓	✓	✓	✓	✓
Social Functioning	✓	✓	✓	✓	✓	✓
Physical Health Summary	✓	✓	✓	✓	✓	✓
Total SF Score	✓	✓	✓	✓	✓	✓
**Total Features Selected**	16	14	17	14	16	14

#### 3.3.1 Chi-squared.

The Chi-Squared test is a frequently used statistical method to check if two categorical variables are significantly related by comparing the observed frequencies to their expected values. The Chi-Squared statistic is calculated using the formula:

$$\chi^{2}=\sum \frac{(O_{i}-E_{i}{)}^{2}}{E_{i}}$$ (1)

Here, *O*_*i*_ is the observed frequency, and *E*_*i*_ denotes the frequency expected for each category, with the summation taken over all categories.

#### 3.3.2 Analysis of variance.

ANOVA is another well-known statistical technique that breaks down data fluctuation to compare group means and determine whether there are significant differences. The ANOVA F-statistic is given as follows:

$$F=\frac{\frac{\sum_{i=1}^{k}n_{i}({\bar{X}}_{i}-\bar{X}{)}^{2}}{k-1}}{\frac{\sum_{i=1}^{k}\sum_{j=1}^{n_{i}}(X_{ij}-{\bar{X}}_{i}{)}^{2}}{N-k}}$$ (2)

In this context, ${\bar{X}}_{i}$ stands for the mean of the *i*-th group, $\bar{X}$ represents the overall average, *n*_*i*_ indicates the sample size of the *i*-th group, *N* refers to the total number of samples, and *k* denotes the number of groups considered.

#### 3.3.3 Mutual information.

MI assesses the reliance of two variables, showing how much information is shared. In feature selection, MI helps identify features with significant information about the target variable. The formula for MI is:

$$I(X;Y)=\sum_{x\in X}\sum_{y\in Y}p(x,y)\log\left(\frac{p(x,y)}{p(x)p(y)}\right)$$ (3)

In equation 3, I(X;Y) gives the information that are mutual between the variables *X* and *Y*. Here, *p*(*x*,*y*) represents the joint probability distribution of both *X* and *Y*, while *p*(*x*) and *p*(*y*) correspond to their respective marginal distributions.

#### 3.3.4 Recursive feature elimination.

RFE selects features by repeatedly removing the least important ones based on model performance until the optimal set is found. The procedure can be represented as:

$$S^{*}=\arg\min_{S}\;\text{Performance}(f(S))$$ (4)

Where S^*^ is the final set of selected features, *f*(*S*) is the model trained on features *S*, and Performance(f(S)) is the model’s performance measure.

#### 3.3.5 Embedded-based (RFE with MI).

This hybrid approach blends filter and wrapper techniques for feature selection. It uses algorithms to identify the most relevant features, which are then fed into the training model. Additionally, embedded feature selection methods are key to improving the process. In this case, we use RFE with Tree-based methods and Gradient-based estimators to pick the most important features.

#### 3.3.6 AMSE-DFI.

Our proposed AMSE-DFI framework is designed to effectively identify and rank features for classification, especially in datasets with diverse data types. It follows three interconnected stages, each adding a unique perspective to the feature selection process.

**Stage 1: Pre-Filtering with Mutual Information** In the first stage, we use a filter-based approach by calculating MI between each feature *X*_*i*_ and the target variable *y*. The following is the definition of mutual information, a metric that measures the dependence between two random variables:

$$MI(X_{i};y)=\sum_{x\in {𝒳}_{i}}\sum_{y\in 𝒴}p(x,y)\log\left(\frac{p(x,y)}{p(x)p(y)}\right)$$ (5)

Features were ranked by MI scores, and the top 70–80% (about 19–22 of 28) were retained, reducing noise and keeping only the most informative predictors for analysis [52].

**Stage 2: Model-Based Ensemble with Recursive Feature Elimination** In the second stage, we combine RFE with an ensemble approach using Random Forest and XGBoost. Random Forest ranks features based on Gini impurity, while XGBoost identifies the most impactful ones by minimizing prediction error. The importance scores from the two models are computed as $I_{\text{RF}}(X_{i})$ and $I_{\text{XGB}}(X_{i})$, respectively. These scores are aggregated using a weighted average:

$$\left(X_{i}{)}_{\text{combined}}=\alpha \cdot I_{\text{RF}}(X_{i})+(1-\alpha )\cdot I_{\text{XGB}}(X_{i}\right)$$ (6)

Where α=0.5 (i.e., equal weighting) in our experiments. The combined importance is then used within an RFE framework, which iteratively eliminates the least important features until a predefined performance threshold (AUC > 0.85) is met [53]. The output of this stage is a refined subset $F_{\text{RFE}}$ containing approximately 10–15 features.

**Stage 3: Dynamic Feature Interaction with Graph Attention Networks** Traditional ensemble methods treat features as independent, but many datasets have complex interdependencies that impact model performance. To address this, we introduce a Graph Attention Network (GAT) to capture these interactions. The following are the key steps involved in the GAT architecture.

**I. Graph Construction**

Each feature in the refined subset $F_{\text{RFE}}$ is represented as a node in an undirected graph G=(V,E). The edge between any two features *X*_*i*_ and *X*_*j*_ is weighted based on their correlation or MI, forming the basis for the connectivity matrix.

**II. GAT Formulation**

The GAT layer computes attention coefficients *a*_*ij*_ for each edge, which quantify the importance of the interaction between nodes *i* and *j*. The attention mechanism is given by:

$$e_{ij}=\text{LeakyReLU}\left(a^{T}\left[Wx_{i}∥Wx_{j}\right]\right)a_{ij}=\frac{\sum_{k\in N(i)}\exp(e_{ik})}{\exp(e_{ij})}$$ (7)

Here, *W* stands for a trainable weight matrix, *a* represents a trainable attention vector, ∥ indicates concatenation, and *N*(*i*) refers to the collection of neighboring nodes associated with node *i*.

**III. Feature Importance Adjustment**

The final GAT-derived feature importance $I_{\text{GAT}}(X_{i})$ is computed by averaging the attention scores across heads and neighboring interactions:

$$I_{\text{GAT}}(X_{i})=\frac{1}{\left|N(i)\right|}\sum_{j\in N(i)}a_{ij}$$ (8)

**IV. Final Aggregation**

The ultimate feature importance score for each feature *X*_*i*_ is derived by combining the scores from all three stages through a weighted ensemble approach:

$$I_{\text{final}}(X_{i})=\max_{j}[0.3\cdot \text{MI}(X_{j};y)+0.4\cdot I_{\text{combined}}(X_{j})+0.3\cdot I_{\text{GAT}}(X_{j})]\;\cdot [0.3\cdot \text{MI}(X_{i};y)+0.4\cdot I_{\text{combined}}(X_{i})+0.3\cdot I_{\text{GAT}}(X_{i})]$$ (9)

This normalization makes the final scores comparable across features. The weights (30% for MI, 40% for the ensemble model, and 30% for the GAT score) are set through cross-validation and sensitivity analysis to enhance predictive performance.

#### 3.3.7 AMSE-DFI model algorithm and feature selection.

Our AMSE-DFI model (Algorithm 1) integrates multiple techniques to select the most important features for the ML model. In Algorithm 1 (Stage 1), the threshold *θ* is set to the 70th percentile of MI scores, to retain the top-k features with the strongest univariate relevance to the mental health target [52]. This value was empirically determined through a sensitivity analysis on the validation subset, evaluating 5-fold cross-validated F1-scores on a baseline LGBM model across percentiles from 50% to 90% in 10% increments. The 70% threshold yielded the optimal balance, retaining ∼20 of 28 features while achieving a cross validation F1-score of 0.98, avoiding aggressive pruning (e.g., 50-60% dropped F1 to ∼0.95 by discarding subtle predictors like Role_Emotional) and excessive noise (80-90% caused minor overfitting with plateaued F1 at 0.97 and higher variance). This approach follows standard MI-based tuning, like scikit-learn’s SelectPercentile, ensuring robust feature selection. Then, it uses two powerful models—Random Forest and XGBoost—to rank the remaining features based on how useful they are, refining them further with a method called RFE. After that, it applies a GAT to understand how the features interact with each other in a more complex way. Finally, it blends the results from all these steps to come up with a final list of the best features to use.

**Algorithm 1 AMSE-DFI algorithm for feature selection.**

1: **function** AMSE-DFI

2:   **Step 1: MI PreFiltering**

3:   Compute MI between each feature *X*_*i*_ and the target *y*.

4:   **Threshold Calculation:**

5:   Compute a threshold percentile *θ* for feature selection (e.g., top 70% percentile).

6:   The threshold can be based on sensitivity analysis.

7:   Select features where $MI(X_{i};y)>\theta$ to form the feature set *F*_*MI*_.

8:   **Step 2: RFC-XGBoost Ensemble RFE**

9:   Train a RFC and an XGBoost classifier.

10:  Calculate feature importances for both RFC and XGBoost models.

11:  Combine RFC and XGBoost feature importances into an ensemble score.

12:  Apply RFE to refine and select the top features, forming *F*_*RFE*_.

13:  **Step 3: GAT Feature Importance**

14:  Construct a feature graph and compute attention weights using a graph attention mechanism.

15:  Compute feature importance from attention scores to form *I*_*GAT*_.

16:  **Step 4: Final Feature Integration**

17:  Combine the feature importance scores from:

18:   • Mutual Information (*I*_*MI*_),

19:   • Ensemble RFC-XGBoost (*I*_*Ensemble*_),

20:   • Graph Attention (*I*_*GAT*_).

21:  Select the top features based on the final combined importance scores to form *F*_*Final*_.

22:  **return** *F*_*Final*_

23: **end function**

**Fig 3:**
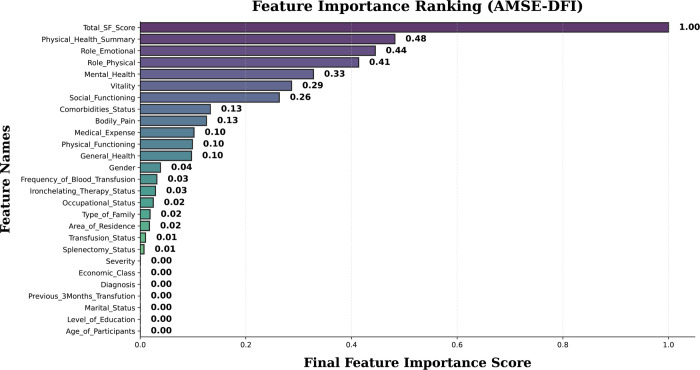
Feature importance plot based on AMSE-DFI for Thalassemia related mental health patients.

Fig 3 visualizes feature importance using the AMSE-DFI method, where features are ranked based on their contribution to the model. The Total_SF_Score stands out as the most significant feature with a score of 1.00, followed by Physical_Health_Summary (0.48), Role_Emotional (0.44), and Role_Physical (0.41). Other features like Social_Functioning (0.29), Comorbidities_Status (0.13), and Bodily_Pain (0.13) also play a noticeable role. Attributes such as Medical_Expense, Physical_Functioning, and General_Health have a smaller impact with a score of 0.10, while Gender (0.04) and Frequency_of_Blood_Transfusion (0.03) contribute only marginally. Features including Severity, Economic_Class, Diagnosis, Marital_Status, Level_of_Education, and Age_of_Participants were identified as having no significant influence on the model’s predictions.

### 3.4 ML model selection

Here, we use ten well-known classification algorithms to predict mental health outcomes in Thalassemia patients. These include Random Forest Classifier (RFC), Extreme Gradient Boosting (XGB), K-Nearest Neighbors (KNN), Support Vector Classifier (SVC), AdaBoost (ADA), LGBM, Artificial Neural Network (ANN), Multilayer Perceptron (MLP), along with two hybrid approaches: PSO based LGBM and PSO based ADA. LGBM and ADA outperformed other models, so we further optimized them using PSO to achieve improved performance. These algorithms were chosen because they are commonly used in similar research, making them reliable and easy to apply [28,54]. Each model was initially implemented with standard configurations. The baseline ML models were manually fine-tuned through a trial-and-error approach to enhance their performance. Additionally, ADA and LGBM models were further optimized using PSO, which automatically identified the most effective hyperparameters for achieving optimal results. Our goal is to identify the classification method that performs best and generalizes most effectively for predicting mental health in Thalassemia patients. The following hyperparameter section details the final configurations used for each model during implementation.

### 3.5 Hyperparameter tuning

After model selection, eight baseline models were fine-tuned through trial-and-error, while PSO-ADA and PSO-LGBM used automated optimization to maximize performance. Table 4 presents the final hyperparameter configurations that achieved optimal predictive efficiency across all experimental tasks. Among the baseline models, LGBM consistently emerged as the top performer, configured with a random state of 42 and 100 estimators. In the PSO-based LGBM model, we tuned key hyperparameters such as the number of estimators (between 10 and 200), learning rate (from 0.01 to 1.0), and maximum tree depth (ranging from 3 to 15). To guide the optimization process effectively, we used a swarm size of 10 and set the number of iterations to 20.

**Table 4:** Model name and hyperparameter optimization.

Model Name	Hyperparameter Optimization
RFC	random_state=42, criterion=’gini’, n_estimators=100
XGB	random_state=42, booster=’gbtree’, n_estimators=100
KNN	n_neighbors=5, weights=’uniform’
SVC	probability=True, C=1.0
AdaBoost	random_state=42, n_estimators=50
LGBM	random_state=42, n_estimators=100
ANN	Loss: Binary Crossentropy, Optimizer: Adam, epochs=100, batch size=32
MLP	Loss: Binary Crossentropy, Optimizer: Adam, epochs=100, batch size=32
PSO-ADA	n_estimators:[10, 200], learning_rate:[0.01, 1.0] swarmsize=10, maxiter=20
PSO-LGBM	n_estimators:[10, 200], learning_rate:[0.01, 1.0] max_depth: [3, 15], swarmsize=10, maxiter=20

### 3.6 Addressing class imbalance through SMOTE-based data resampling

To address the class imbalance in our mental health prediction model, we applied oversampling to increase the minority class instances. By combining the minority class instance with its nearest neighbors, the SMOTE generates synthetic samples [55]. This helps balance the dataset by generating new, similar data points. The SMOTE equation is as follows:

$$\hat{x}=x_{i}+\lambda (x_{k}-x_{i})$$ (10)

In Eq 10, $\hat{x}$ represents the synthetic sample, *x*_*i*_ is the original instance from the minority class, *x*_*k*_ is a randomly chosen neighbor, and λ is a value randomly selected from the interval [0,1] and used to create the new sample.

### 3.7 Evaluation metrics

To evaluate our proposed model, we used key metrics derived from the confusion matrix, including precision, recall, F1-score, accuracy, and AUROC. In binary classification, True Positives (TP), False Positives (FP), True Negatives (TN), and False Negatives (FN) represent correct and incorrect predictions for positive and negative classes. Precision measures positive prediction accuracy, recall reflects true positive detection, and F1-score balances both for imbalanced data. Accuracy shows overall correctness, while a higher AUROC indicates better class discrimination. The corresponding formulas for these metrics are presented below.

$$\text{Precision}=\frac{TP}{TP+FP}$$ (11)

$$\text{Recall}=\frac{TP}{TP+FN}$$ (12)

$$\text{F1-}\mathrm{score}=2\times \frac{\text{Precision}\times \text{Recall}}{\text{Precision}+\text{Recall}}$$ (13)

$$\text{Accuracy}=\frac{TP+TN}{TP+TN+FP+FN}$$ (14)

### 3.8 XAI utilizing LIME

XAI helps make AI systems clearer and easier to understand by showing how decisions are made [56]. In this study, we utilized LIME, a tool that explains AI predictions by showing the contribution from each feature to the model’s decisions, making the process more transparent [57]. LIME was selected over other XAI methods (e.g; SHAP, SHAPASH) as it offers interpretable explanations for individual predictions, aligning with our objective of understanding outcomes in mental health analysis [58,59]. The explanation model for LIME is as follows:

$$\hat{g}\left(x\right)=argmin_{g\epsilon G}L\left(f,g,\pi_{x^{\prime }}\right)+\Omega \left(g\right)$$ (15)

In equation 15, the explanation model $\hat{g}(x)$ uses simpler models, usually linear, to approximate the complex model’s behavior. The loss function $L(f,g,\pi_{x^{\prime }})$ balances accuracy and simplicity, ensuring the explanation is both precise and interpretable.

## 4 Experimental results

We conducted our experiments using Google Colab [60], taking advantage of its stable environment for developing and refining ML models. Its collaborative features streamlined teamwork by allowing easy code and dataset sharing. To fine-tune hyperparameters, we implemented PSO using the pyswarm library [61]. All model development, including LIME explanations [62], was carried out in Python 3 with Scikit-Learn [63]. We tested cross-validation with different fold ranges (1–10) for all final ML model configurations, and experimental results showed that 5-fold cross-validation provided the most stable performance. This finding aligns with previous classification studies reporting 5-fold as the most effective setup [64]. In our analysis, cross-validation was applied at every training stage—both before and after using SMOTE—to ensure a fair and reliable evaluation. This section compares ML models for predicting mental health in Thalassemia patients, highlighting key features using various selection methods, including our novel approach. Oversampling techniques were applied to address class imbalance, and LIME explanations provided insights into feature contributions. To further validate the effectiveness of our proposed framework, we benchmarked its performance against well-established Thalassemia dataset. Experimental results reveal that our proposed model outperforms existing approaches overall, as detailed in the following analysis.

### 4.1 Classification performance with feature selection and SMOTE integration

In this experiment, we evaluated multiple ML and DL models using optimization-based classification across different configurations. Table 5 presents the performance results for models without feature selection, with feature selection, and with feature selection combined with SMOTE. Without feature selection, the PSO-based ADA and PSO-based LGBM models demonstrated strong performance, each achieving 96.26% accuracy. Notably, the ADA model outperformed all others, reaching 97.22% accuracy, with 95% precision, 95% recall, 95% F1-score, and an AUROC of 98%.

**Table 5:** Comprehensive performance comparison of ML models under different configurations.

Configuration	Feature Selection	Model	Accuracy (%)	Precision (%)	Recall (%)	F1-Score (%)	AUROC (%)
Without Feature Selection	None	RFC	90.65	88.00	75.86	81.48	98
		XGB	94.44	94.44	85.00	89.47	98
		KNN	72.22	50.00	25.00	33.33	69
		SVC	73.61	75.00	5.00	10.97	45
		ADA	97.22	95.00	95.00	95.00	98
		PSO-ADA	96.26	96.29	89.65	92.86	98
		LGBM	95.83	94.74	90.00	92.31	98
		PSO-LGBM	96.26	93.10	93.10	93.10	98
		ANN	58.33	40.00	95.00	55.88	72
		MLP	75.00	53.57	75.00	63.00	84
With Feature Selection	Chi-Squared	SVC	94.44	94.44	85.00	89.47	95
		ADA	97.22	98.00	90.00	94.74	97
		PSO-LGBM	97.22	95.00	95.00	95.00	98
		KNN	94.44	94.44	85.00	89.47	93
	ANOVA	ADA	95.83	94.74	90.00	92.31	95
		PSO-LGBM	97.22	98.00	90.00	94.74	98
		PSO-ADA	95.83	90.48	95.00	92.68	96
	MI	XGB	94.44	94.44	85.00	89.47	95
		PSO-LGBM	97.22	97.67	90.00	94.74	97
		PSO-ADA	97.22	98.00	90.00	94.74	98
	RFE	RFC	93.06	94.12	80.00	86.49	94
		PSO-LGBM	97.22	95.00	95.00	95.00	98
		XGB	94.44	94.44	85.00	89.47	96
	RFE-MI	PSO-ADA	97.22	95.00	95.00	95.00	98
		PSO-LGBM	97.22	97.12	90.00	94.74	97
		ADA	97.22	95.00	95.00	95.00	96
	AMSE-DFI	PSO-ADA	98.61	98.37	95.00	97.43	98
		PSO-LGBM	98.61	98.37	95.00	97.43	98
		ADA	95.83	94.74	90.00	92.31	95
With Feature Selection + SMOTE	None	PSO-ADA	95.23	94.32	85.00	91.89	94
		PSO-LGBM	95.33	92.86	89.66	91.23	96
		SVC	95.83	94.74	90.00	92.31	95
	Chi-Squared	ADA	97.22	98.00	90.00	94.74	97
		PSO-LGBM	98.61	98.06	95.00	97.43	98
		KNN	90.28	78.26	90.00	83.72	92
	ANOVA	ADA	97.22	98.00	90.00	94.74	97
		PSO-LGBM	98.61	97.72	95.00	97.43	97
		PSO-ADA	98.61	98.12	95.00	97.43	98
	MI	XGB	95.83	94.74	90.00	92.31	96
		PSO-LGBM	98.61	98.12	95.00	97.43	98
		PSO-ADA	97.22	98.00	90.00	94.74	97
	RFE	RFC	95.83	94.74	90.00	92.31	95
		PSO-LGBM	98.61	98.06	95.00	97.43	98
		XGB	94.44	94.44	85.00	89.47	95
	RFE-MI	PSO-ADA	98.61	98.00	95.00	97.43	98
		PSO-LGBM	98.61	98.12	95.00	97.43	98
		ADA	97.22	98.00	90.00	94.74	97
	AMSE-DFI	PSO-ADA	98.61	98.12	95.00	97.43	98
		PSO-LGBM	**99.07**	**99.00**	**97.78**	**98.88**	**99**

With feature selection, we applied six different methods, including our proposed AMSE-DFI, to identify the most influential features. For each method, we present the top three performing ML models, highlighting the best results achieved with each approach. In Chi-Squared and ANOVA, PSO-Based LGBM achieved the highest accuracy of 97.22%, using 16 and 14 selected features, respectively. Similarly, in MI, with 17 features, PSO-Based LGBM maintained its top accuracy of 97.22%. For RFE and RFE-MI, both PSO-Based ADA and PSO-Based LGBM achieved the highest accuracy of 97.22%, using 14 and 16 features, respectively. Among all the techniques analyzed, our proposed AMSE-DFI method with 14 features, delivered the best performance, with PSO-based ADA and PSO-based LGBM emerging as the top-performing models. Both models achieved the highest accuracy of 98.61%, with a perfect precision score of 98.37%, a recall of 95%, an F1-score of 97.43%, and an AUROC of 98%.

After applying the feature selection methods, we used SMOTE to address class imbalance, and the top three models from each approach are presented in Table 5. Without using feature selection, the ADA model achieved an accuracy of 95.83% after applying SMOTE to balance the data set. However, when we used the five feature selection techniques along with SMOTE, several models performed significantly better, reaching an accuracy of 98.61%. Among them, our proposed AMSE-DFI method, combined with SMOTE, led to the best results. The PSO-based LGBM model achieved an outstanding accuracy of 99.07%, with a precision of 99%, recall of 97.78%, F1-score of 98.88%, and an AUROC score of 99%.

The AMSE-DFI framework outperforms traditional feature selection methods by combining multi-stage, adaptive strategies that capture both individual relevance and dynamic feature interactions. Unlike univariate methods (e.g., MI, F1-score 97.43%) or wrapper-based methods (e.g., RFE, F1-score 97.43%) that consider features independently or linearly, AMSE-DFI integrates a Graph Attention Network to model non-linear dependencies in the heterogeneous SF-36 data. This produces a highly discriminative 14-feature subset, leading to superior performance with an F1-score of 98.88% and an AUROC of 99%. The weighted fusion of MI (30%), RFE (40%), and GAT (30%) balances relevance, redundancy, and interactions, making AMSE-DFI more robust to small, imbalanced datasets and highly effective for predicting mental health outcomes in Thalassemia patients.

**Fig 4:**
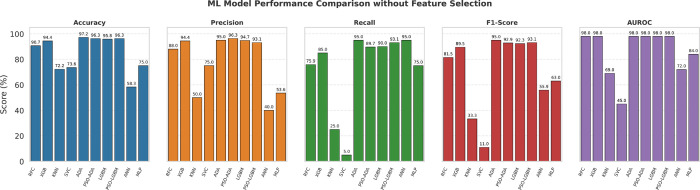
Performance comparison of ML and DL models without feature selection.

**Fig 5:**
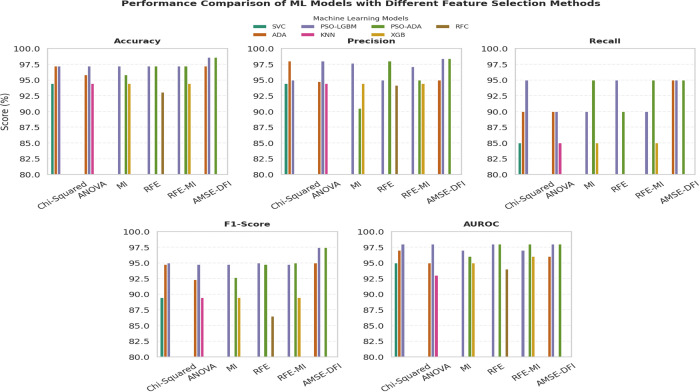
Performance comparison of ML models using different feature selection methods.

**Fig 6:**
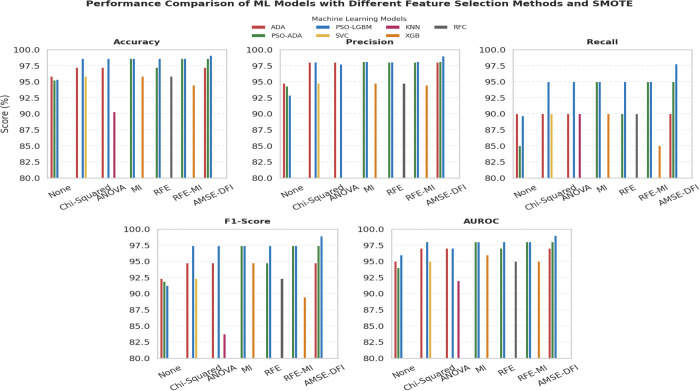
Performance comparison of ML models using different feature selection methods with SMOTE.

Figs 4, 5, and 6 present graphical bar chart comparisons corresponding to the results summarized in Table 5. Fig 4 illustrates the performance of eight ML and DL models without feature selection. Fig 5 compares the top three performing models for each feature selection method, while Fig 6 visualizes the top three models after applying both feature selection and SMOTE. Each figure displays a comparative analysis of accuracy, precision, recall, F1-score, and AUROC to highlight model performance under different configurations.

**Fig 7:**
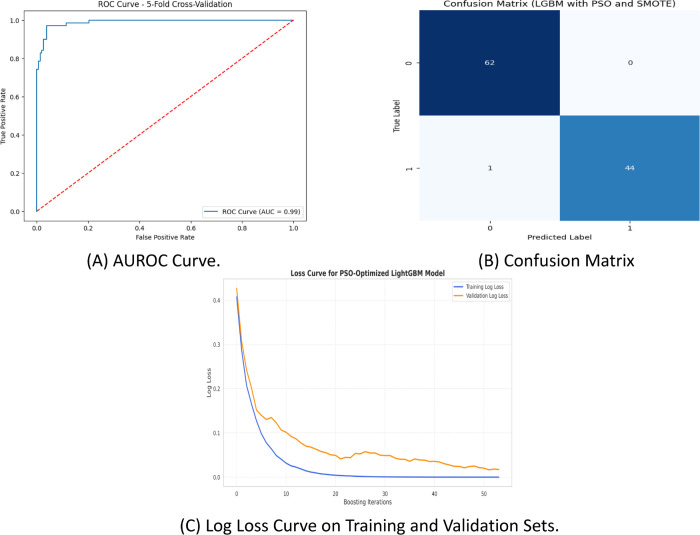
(A) AUROC curve, (B) confusion matrix, and (C) log loss curve for the PSO-based LGBM model with SMOTE and AMSE-DFI feature selection.

Fig 7 illustrates the AUROC curve, confusion matrix, and log loss curve for the PSO-based LGBM model enhanced with SMOTE and feature selection using AMSE-DFI. Fig 7 A shows an AUROC score of 99% for the PSO-based LGBM model with SMOTE, indicating a flawless classification with the curve. In Fig 7 B, the confusion matrix highlights 62 true negatives, 44 true positives, 1 false negative, and no false positives, reflecting excellent accuracy with minimal errors. Fig 7 C illustrates the log loss for both the training and validation sets across boosting iterations. Both curves consistently drop, with the validation loss stabilizing at a low value and showing no signs of divergence. This suggests strong generalization and effective regularization, confirming that the model is well-balanced without over- or under-fitting.

### 4.2 Statistical significance analysis

To ensure the robustness of our findings, we conducted statistical significance tests across key metrics (Accuracy, Precision, Recall, F1-score, and AUROC) using 5-fold cross-validation results. Since all models used the same data splits, we applied the Wilcoxon signed-rank test for paired comparisons, the Friedman test with Nemenyi post-hoc analysis for overall model comparison, and McNemar’s test to evaluate differences in misclassification rates.

Table 6 shows that the proposed AMSE-DFI with PSO-LGBM model demonstrated strong statistical superiority and robustness. It achieved the highest F1-score of 98.88%, outperforming the next-best model PSO-ADA with AMSE-DFI, F1 97.43%) with a Wilcoxon *p*-value of 0.008, confirming significant improvement. Both AUROC and Accuracy also showed notable gains (*p*<0.01) over traditional feature selection methods (RFE, MI, Chi-squared, ANOVA). McNemar’s test ($\chi^{2}$ 9.84, *p* 0.002) indicated fewer misclassification errors, while the Friedman test ($\chi^{2}$ 18.7, *p*<0.001) confirmed the model’s top-ranked performance across all comparisons.

**Table 6:** Summary of statistical significance tests for top-performing models.

Comparison	Metric	Test	Statistic	p-value
AMSE-DFI + PSO-LGBM vs PSO-ADA + AMSE-DFI	F1-score	Wilcoxon	12.5	0.008
AMSE-DFI + PSO-LGBM vs PSO-LGBM (RFE)	AUROC	Wilcoxon	14.0	0.005
AMSE-DFI + PSO-LGBM vs PSO-LGBM (No Feature Selection)	Accuracy	Wilcoxon	11.2	0.012
AMSE-DFI + PSO-LGBM vs PSO-LGBM (No Feature Selection)	Confusion Matrix	McNemar	9.84	0.002
Top 3 models across all Feature Selection methods	F1-score	Friedman	18.7	<0.001

### 4.3 Key feature relationships and insights

**Fig 8:**
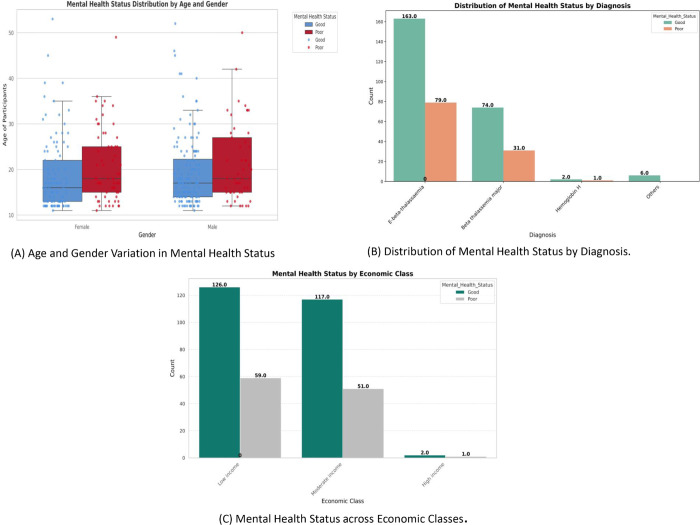
Multi-dimensional analysis of mental health status across demographic, clinical, and socioeconomic factors — (A) Age and gender variation, (B) Diagnosis-based distribution, and (C) Economic class comparison.

This section explores the relationship between the mental health status of Thalassemia patients and various factors, including demographic, clinical, economic, healthcare, and functional perspectives. Fig 8 highlights the three important features influencing the mental health of Thalassemia patients. As shown in Fig 8 A, younger participants (particularly aged 13–22) exhibit better mental health compared to older age groups, suggesting that mental well-being tends to decline with age due to prolonged disease burden and psychosocial stress. Females show slightly higher rates of poor mental health (38.04%) than males (25.39%), possibly reflecting gender-based differences in emotional resilience and social support. Fig 8 B reveals that patients with less severe Thalassemia types (e.g., E-*β* thalassemia) demonstrate higher mental well-being than those with *β* thalassemia major, indicating that disease severity and treatment intensity directly affect psychological outcomes. Fig 8 C shows a positive link between income and mental health, where low- and moderate-income groups report higher distress, highlighting the influence of financial constraints on treatment accessibility and quality of life.

**Fig 9:**
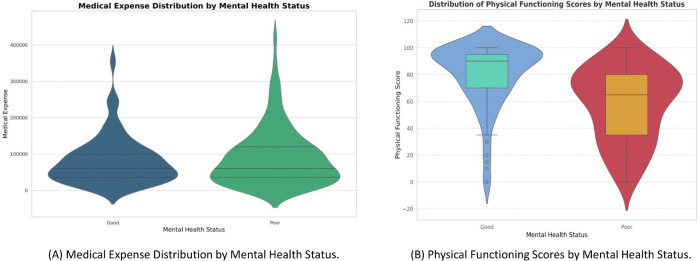
Multi-dimensional analysis of mental health status across healthcare and functional dimensions — (A) Medical expense distribution by mental health status, and (B) Physical functioning score comparison.

Fig 9 presents a multi-dimensional analysis of mental health status using violin plots, highlighting healthcare and functional perspectives by focusing on two important features, medical expenses and physical functioning scores. Fig 9 A compares annual medical expenses between individuals with ’Good’ (blue) and ’Poor’ (green) mental health. Patients with lower medical expenses (below 100,000) are more likely to report good mental health, while higher expenses are associated with poorer outcomes, indicating that financial burden from treatment may negatively impact mental well-being. Fig 9 B shows physical functioning scores, revealing that individuals with good mental health generally have higher and more varied scores (median ∼70), whereas those with poor mental health show lower physical functioning (median ∼50). This suggests a strong link between physical capacity and psychological well-being, where decreased functioning may exacerbate stress, anxiety, or depressive symptoms. Together, these findings highlight how both economic and functional factors critically influence mental health in Thalassemia patients.

### 4.4 Using LIME to interpret predictions of proposed model

**Fig 10:**
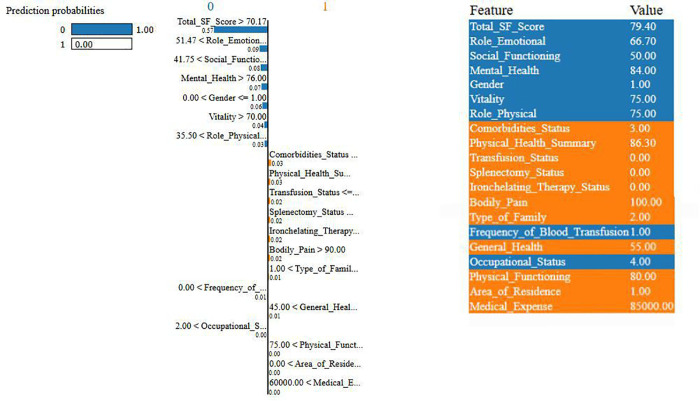
LIME tabular explainer plot for predicting mental health status in Thalassemia patients.

**Fig 11:**
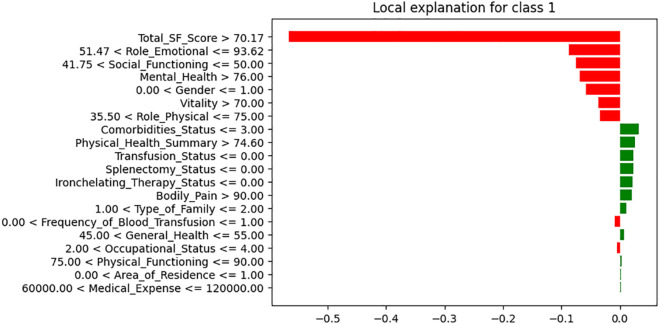
LIME feature importance plot for predicting mental health status in Thalassemia patients.

We are analyzing features using XAI techniques as part of our proposed framework. In this analysis, LIME does a great job of highlighting both the positive and negative impactful features through its tabular explainer and feature importance plots. Figs 10 and 11 show LIME explanations for a representative instance interpreting mental health prediction. This approach reflects LIME’s core purpose of explaining how individual features influence a specific model prediction. Fig 10 shows the LIME tabular plot, highlighting feature influences on the model’s prediction, generated dynamically for that specific observation. Key factors, like total SF scores, role-emotional metrics, and mental health scores, can push the prediction toward a more positive or negative outcome. For example, higher SF and mental health scores (e.g., >70.17 and 76.0) lead to a more favorable prediction, while there is a decrease in the likelihood of a particular result as in for aspects like Comorbidities_Status and Physical_Health_Summary. The table on the right of Fig 10 shows the exact values of these features, whereas the bar representation given in the left side of the figure reflects the final prediction probabilities. Fig 11 shows the LIME feature importance plot, highlighting why the model predicts an instance as class 1 (poor condition). Features shown in red increase the likelihood of this classification, while those in green decrease it. Factors linked to poor health include Total_SF_Score <70.17, Role_Emotional <93.62, Social_Functioning <50, and Mental_Health <76. Having three or fewer comorbidities lowers the risk, while a Physical_Health_Summary of <74.60 unexpectedly reduces the likelihood of poor health classification. Across multiple samples, both plots showed consistent patterns, with the same features repeatedly emerging as key predictors. This indicates that while LIME explanations are local, their feature impact trends remain consistent across the dataset.

### 4.5 Benchmarking model performance on existing dataset

While several studies have explored Thalassemia prediction, few have examined patients’ mental health. To address this, we used a new feature-based dataset to analyze factors affecting their mental well-being. Table 7 compares the methods and prediction accuracy of previous studies with those of our proposed approach for Thalassemia mental health. In the study by Saleem et al. [39], a Gradient Boosting Classifier (GBC) model combined with SMOTE and feature selection achieved 93.46% accuracy for Thalassemia prediction. Since mental health Thalassemia datasets are private, we performed external validation using their dataset. Our AMSE-DFI feature selection identified seven key features, and with SMOTE and a PSO-based RFC model, we achieved 94.29% accuracy, 93.88% precision, 94.33% recall, 94.04% F1-score, and an AUROC of 99%. This demonstrates its efficiency and robustness in predicting mental health in Thalassemia patients.

**Table 7:** Comparison of methods and accuracy for Thalassemia prediction.

Study	Data size	Method	Features	Accuracy (%)
[26]	1260	SVM, NB, KNN, DT, RF, PCA	27 features	82.5
[31]	310	MLR, MLP, SHAP, LIME	12 features	97.81
[39]	616	GBC with SMOTE and feature importance-selection	13 features	93.46
[32]	350	Support Vector Machine	24 features	76.0
[38]	1259	LR, KNN, RF, DT, Stacking Regression	27 features	81.75
Our study	356	Proposed AMSE-DFI with SMOTE and PSO-LGBM	14 features	99.07

## 5 Discussion

In the literature review, we explore how AI and ML have been used to diagnose and assess mental health in Thalassemia patients, focusing on classification methods and risk prediction, as shown in Table 1. Earlier studies [31,33,37–40,42] often missed key aspects such as proper feature selection, balanced data, and important variables considered in our study. To overcome these gaps, we present a new feature selection framework that aims to make mental health prediction more accurate and meaningful. We compared standard feature selection methods with our proposed AMSE-DFI model, which achieved superior accuracy. Using 10 ML models, including DL and PSO-based optimization, we analyzed the Mendeley dataset with SMOTE balancing. Model performance was evaluated using Accuracy, Precision, Recall, F1-score, and AUROC. In AMSE-DFI, Total_SF_Score (1.00) was the most influential feature, followed by Physical_Health_Summary (0.48), Role_Emotional (0.44), and Role_Physical (0.41), while features like Severity, Economic_Class, and Age showed minimal impact. Normalized feature weights were optimized via cross-validation with proportions of 30% MI, 40% Ensemble, and 30% GAT. Features like Total_SF_Score, Physical_Health_Summary, and Role_Emotional stood out because they capture the overall picture of a patient’s well-being—both physically and emotionally. These measures reflect how daily functioning, energy, and emotional balance are closely tied to mental health. In our model, they naturally carried more weight since they summarize a person’s quality of life rather than a single symptom. The AMSE-DFI framework also recognized how these factors interact, showing that good physical health and emotional strength often go hand in hand with better mental well-being in Thalassemia patients.

Table 5 presents the performance of different model configurations including without feature selection, with feature selection, and with feature selection plus SMOTE. Initially, without feature selection, the ADA model performed best, achieving 97.22% accuracy, 95% precision, recall, and F1-score, and an AUROC of 98%. To further evaluate performance, we applied five standard feature selection methods, which maintained a top accuracy of 97.22%. In contrast, our custom AMSE-DFI feature selection significantly improved results, with both PSO-based ADA and PSO-based LGBM reaching 98.61% accuracy, 98.37% precision, 95% recall, 97% F1-score, and AUROC of 98%. After balancing the dataset with SMOTE and applying all feature selection techniques, our proposed approach with PSO-based LGBM achieved the highest performance, reaching 99.07% accuracy, 99% precision, 97.78% recall, and 98.88% F1-score. With a stable AUROC of 99% and very few misclassifications, the proposed model demonstrates strong generalization. Its consistently decreasing training and validation log loss shows effective regularization, confirming that the model is well-balanced and free from overfitting or underfitting.

We examined key features influencing Thalassemia patients’ mental health and found that multiple factors affect their overall psychological health. Men generally fare better than women, and younger patients tend to have healthier mental states compared to older ones. The type of Thalassemia also matters that patients with E-*β* thalassemia and *β* thalassemia major often report better mental health. Additionally, those with low to moderate incomes seem to enjoy more favorable mental health, while higher medical expenses are linked to poorer outcomes. Lastly, higher physical functioning scores are closely associated with improved mental health.

The XAI LIME analysis in our study clarifies the specific contributions of key SF-36 features—such as Total_SF_Score, Role_Emotional, and Physical_Health_Summary—to predicting mental health in Thalassemia patients. For example, higher Role_Emotional scores reduce predicted distress by up to 25% in local explanations, whereas lower Physical_Health_Summary values increase anxiety risk through complex interactions with demographic factors like age and gender. These insights support personalized clinical interventions: patients with low Role_Emotional scores may benefit from cognitive-behavioral therapy (CBT) focused on emotional coping and social engagement, while deficits in Physical_Health_Summary highlight the need for integrated care, including tailored iron chelation adherence and fatigue management programs to break the fatigue-depression cycle observed in over one-third of adults. By revealing both protective effects (e.g., high Total_SF_Score) and risk factors (e.g., gender-specific emotional role challenges), LIME enables evidence-based, individualized psychosocial support, improves treatment adherence, and enhances mental health outcomes, while uncovering subtle predictors that traditional assessments often miss.

We benchmarked our approach against previous mental health studies using a well-established Thalassemia dataset. While earlier work achieved 93.46% accuracy, our AMSE-DFI framework with SMOTE and PSO-based RFC reached 94.29%, surpassing prior methods and demonstrating its potential for more accurate mental health prediction in Thalassemia patients. Several limitations of our framework should be noted. First, the dataset was relatively small (n = 356), which may limit the generalizability of our findings despite using cross-validation and SMOTE to balance the classes. Access to a larger and more diverse dataset would allow for a more robust evaluation and further reduce the risk of overfitting. Second, the data were collected exclusively from Bangladeshi Thalassemia patients, which may reflect cultural, socioeconomic, and healthcare-specific factors that differ from other populations, potentially limiting broader applicability.

Clinically, this framework offers a practical decision-support tool for healthcare professionals caring for Thalassemia patients. By highlighting key predictors, it helps clinicians quickly identify individuals at higher risk of psychological distress using routine health assessments. The XAI component (LIME) builds trust by clearly showing the reasoning behind each prediction, allowing doctors, psychologists, and counselors to interpret results intuitively. Integrating this system into hospital electronic health records or screening platforms could enable continuous monitoring of patients’ mental health alongside physical health, supporting earlier interventions, personalized counseling, and more comprehensive, holistic care.

## 6 Conclusions and future works

In this study, we introduce a new framework for predicting the mental health of Thalassemia patients using the SF-36 questionnaire. By combining our custom AMSE-DFI feature selection with XAI techniques such as LIME and enhancing our models with SMOTE and PSO-based optimization, using models such as RFC and LGBM, we achieved outstanding performance with accuracies reaching up to 99.07%. Our approach not only outperforms existing state-of-the-art methods, it also provides clear insights into key factors like overall SF scores, role-emotional metrics, and physical health measures. Our framework ensures reliable and interpretable mental health prediction through robust feature selection and balanced sampling, showing strong potential for broader healthcare applications.

In future research, the AMSE-DFI framework will be expanded to larger, multi-center datasets to validate its scalability, dependability, and generalization across diverse populations, ensuring robustness beyond the Bangladeshi cohort. The approach will also be extended to multi-modal datasets that combine laboratory data with psychological surveys, enabling a more holistic understanding of patient well-being. Although AMSE-DFI is primarily designed for tabular data, its methodology could theoretically be adapted to multi-modal inputs—such as pre-extracted embeddings from medical images or digital health records—after applying appropriate feature extraction methods like CNN or transformer-based encoders, which will be explored in future experiments. Additionally, AMSE-DFI will be evaluated on longitudinal mental health datasets to capture temporal variations in psychological states, facilitating early detection and intervention. Further investigations will explore additional optimization and explainability strategies, including swarm intelligence variants, Bayesian optimization, and advanced XAI techniques such as SHAP and Shapash, to enhance interpretability, stability, and clinical applicability.

## References

[1] Sari TT, Rahmartani LD, Wirahmadi A, Selene NB, Iskandar SD, Wahidiyat PA. Psychological burden among pediatric thalassemia major patients in Indonesia: A review. Thalassemia Rep. 2024;14(2):33–43. doi: 10.3390/thalassrep14020005

[2] Tuo Y, Li Y, Li Y, Ma J, Yang X, Wu S, et al. Global, regional and national burden of thalassemia 1990 -2021: A systematic analysis for the global burden of disease study 2021. EClinicalMedicine. 2024;72:102619. doi: 10.1016/j.eclinm.2024.102619 38745964 PMC11090906

[3] Goh LPW, Chong ETJ, Lee P-C. Prevalence of alpha (*α*)-Thalassemia in Southeast Asia (2010-2020): A meta-analysis involving 83,674 subjects. Int J Environ Res Public Health. 2020;17(20):7354. doi: 10.3390/ijerph17207354 33050119 PMC7600098

[4] El-Beshlawy A, Dewedar H, Hindawi S, Alkindi S, Tantawy AA, Yassin MA, et al. Management of transfusion-dependent β-thalassemia (TDT): Expert insights and practical overview from the Middle East. Blood Rev. 2024;63:101138. doi: 10.1016/j.blre.2023.101138 37867006

[5] Ayon SS, Hossain ME, Miah MSU, Arafin BMM, Chowdhury A, Prova NNI. Thalassemia dataset covering clinical, socioeconomic, and mental health aspects. Data Brief. 2025;63:112082. doi: 10.1016/j.dib.2025.112082 41089732 PMC12516501

[6] Yousuf R, Akter S, Wasek SM, Sinha S, Ahmad R, Haque M, et al. Thalassemia: A review of the challenges to the families and caregivers. Cureus. 2022;14(12). 10.7759/cureus.32491 PMC9747324 36523854

[7] Mardhiyah A, Mediani HS, Panduragan SL, Yosep I, Lindayani L. Hope and quality of life among adolescent with Thalassemia: A cross-sectional study in Indonesia. Open Access Maced J Med Sci. 2022;10(G):667–73. doi: 10.3889/oamjms.2022.9597

[8] Wangi K, Shaleha R, Wijaya E, Birriel B. Psychosocial problems in people living with Thalassemia: A systematic review. SAGE Open Nurs. 2025;11:23779608251323811. doi: 10.1177/23779608251323811 40114814 PMC11924095

[9] Tarım HS, Öz F. Thalassemia major and associated psychosocial problems: A narrative review. Iran J Public Health. 2022;51(1):12–8. doi: 10.18502/ijph.v51i1.8287 35223621 PMC8837879

[10] Sharma SK, Alutaibi AI, Khan AR, Tejani GG, Ahmad F, Mousavirad SJ. Early detection of mental health disorders using machine learning models using behavioral and voice data analysis. Sci Rep. 2025;15(1):16518. doi: 10.1038/s41598-025-00386-8 40360580 PMC12075568

[11] Maryam M, Rahman A, Haider S. Investigation of the relationship between Thalassemia and depression to predict a base for rehabilitation measures. J Disability Res. 2024;3(5). doi: 10.57197/jdr-2024-0045

[12] Jamali AA, Berger C, Spiteri RJ. Identification of depression predictors from standard health surveys using machine learning. Curr Res Behav Sci. 2024;7:100157. doi: 10.1016/j.crbeha.2024.100157

[13] Sener D, Sadri S, Gursoy V. Investigation of depression, anxiety, sleep quality, and fatigue in Thalassemia major patients: A study of the correlation between sleep quality and laboratory findings. Cureus. 2024;16(10):e72614. doi: 10.7759/cureus.72614 39610629 PMC11604234

[14] Mediani HS, Fuadah NT. Factors contributing to anxiety in adolescents surviving thalassemia major in Indonesia. BMC Pediatr. 2025;25(1). doi: 10.1186/s12887-025-05403-3 PMC11783949 39891063

[15] Pan J, Xu A, Tung T-H, Shen B. Investigation on the anxiety and depression status of Thalassemia carriers after genetic counseling. Neuropsychiatr Dis Treat. 2025;21:2357–69. doi: 10.2147/NDT.S549787 41141563 PMC12553394

[16] Zafar M. Common mental disorders and its associated factors among Thalassemic patients. Ann Indian Psychiatry. 2022;6(4):328–31. doi: 10.4103/aip.aip_44_22

[17] Aguirre SI, Ornelas M, Blanco H, Jurado-García PJ, Benavides EV, Rodríguez-Villalobos JM, et al. Quality of life in Mexican older adults: Factor structure of the SF-36 questionnaire. Healthcare (Basel). 2022;10(2):200. doi: 10.3390/healthcare10020200 35206815 PMC8872441

[18] ALMahadin G, Abu Owida H, Al Nabulsi J, Turab N, Al Hawamdeh N. Automated detection of kidney masses lesions using a deep learning approach. IJ-AI. 2024;13(3):2862. doi: 10.11591/ijai.v13.i3.pp2862-2869

[19] Altayeb M, Arabiat A. Enhancing stroke prediction using the waikato environment for knowledge analysis. IJ-AI. 2024;13(3):3010. doi: 10.11591/ijai.v13.i3.pp3010-3017

[20] Hossain MJ, Islam MW, Munni UR, Gulshan R, Mukta SA, Miah MS. Health-related quality of life among thalassemia patients in Bangladesh using the SF-36 questionnaire. 2024. https://data.mendeley.com/datasets/7c2zd56mzd/2 10.1038/s41598-023-34205-9 PMC10182078 37173392

[21] Atlam E-S, Rokaya M, Masud M, Meshref H, Alotaibi R, Almars AM, et al. Explainable artificial intelligence systems for predicting mental health problems in autistics. Alexandria Eng J. 2025;117:376–90. doi: 10.1016/j.aej.2024.12.120

[22] Drahos J, Boateng-Kuffour A, Calvert M, Levine L, Dongha N, Li N, et al. Health-related quality-of-life impacts associated with transfusion-dependent β-Thalassemia in the USA and UK: A qualitative assessment. Patient. 2024;17(4):421–39. doi: 10.1007/s40271-024-00678-7 38530509 PMC11189963

[23] Asim S, Naqvi S, Soomro MA, Karmani VK, Mehmood M, Sharif M, et al. Anxiety, depression, and perceived social support in patients with transfusion dependent beta Thalassemia major. J Liaquat Univ Med Health Sci. 2023;22(04):288–294. doi: 10.22442/jlumhs.2023.02204.213

[24] Mushtaq M, Imtiaz E, Sarfraz A, Rafia Rahat S. Examining the complex interplay of pain perception, pain anxiety, and mental health problems in Thalassemia patients: A mediational analysis. NNJP. 2024;4(1):22–30. doi: 10.53107/nnjp.v4i1.74

[25] Vadakkiniath IJ, et al. Prevalence and correlates of stress, anxiety, and depression in patients with chronic diseases: A cross-sectional study. Middle East Curr Psychiatry. 2023;30(1). doi: 10.1186/s43045-023-00340-2

[26] Sharma M, Mahapatra S, Shankar A, Wang X. Predicting the utilization of mental health treatment with various machine learning algorithms. Mental Health. 2021;1(2):3.

[27] Alhaj Mohammad AA, Alhajji ET, Kadri SA, Alhaj MN. A study of the widespread of anxiety, stress and depression of thalassemia patients among thalassemia centers in Damascus; 2022.

[28] Ayon SS, Hossain ME, Miah MSU, Rahman MM, Mahmud M. Advancing mental health problems with machine learning and genetic algorithms for anxiety classification in Bangladeshi university students. In: International conference on brain informatics. Springer; 2024. p. 338–50.

[29] Hiradfar AA, Noorazar SG. Frequency of psychological disorders in under 15 years old children with thalassemia major and intermediate in Tabriz children’s hospital. Med J. 2021;42(6):730–6. doi: 10.34172/mj.2021.014

[30] Adly A, Ismail E, Salah N, Ibrahim G, Ahmed S. P1462: Neurocognitive function and psychological assessment in transfusion-dependent beta-thalassemia patients: Relation to brain iron content. HemaSphere. 2023;7(S3):e185302c. doi: 10.1097/01.hs9.0000972732.18530.2c

[31] Humayun A, Nawi MABA, Siddiqui MI, Kabir R, Babalola A. A hybrid mathematical framework combining logistic regression and neural networks with explainable AI techniques for mental health prediction. Contemp Math. 2025; p. 6521–40. 10.37256/cm.6520258031

[32] Fu Y-K, Liu H-M, Lee L-H, Chen Y-J, Chien S-H, Lin J-S, et al. The TVGH-NYCU Thal-classifier: Development of a machine-learning classifier for differentiating Thalassemia and non-Thalassemia patients. Diagnostics (Basel). 2021;11(9):1725. doi: 10.3390/diagnostics11091725 34574066 PMC8467438

[33] Basu D, Sinha R, Sahu S, Malla J, Chakravorty N, Ghosal PS. Identification of severity and passive measurement of oxidative stress biomarkers for β–thalassemia patients: K-means, random forest, XGBoost, decision tree, neural network based novel framework. Adv Redox Res. 2022;5:100034. doi: 10.1016/j.arres.2022.100034

[34] Al-Hakeim HK, Najm AH, Moustafa SR, Maes M. Construction of an exposure-pathway-phenotype in children with depression due to transfusion-dependent thalassemia: Results of (un)supervised machine learning. J Affect Disord. 2021;282:644–55. doi: 10.1016/j.jad.2020.12.089 33445087

[35] Bharath M, Gowtham S, Kodipalli A, Rao T. Enhancing alpha Thalassemia screening: A comparative study of multiple machine learning classifiers and interpretation using explainable AI. In: 2023 4th international conference on intelligent technologies (CONIT); 2024. p. 1–7. 10.1109/conit61985.2024.10626908

[36] Wiratchawa K, Petiwathayakorn T, Srichairatanakool S, Koonyosying P, Jarujareet U, Intharah T. ThalNet: Deep learning for Thalassemia via blood image structure function image. In: 2024 international technical conference on circuits/systems, computers, and communications (ITC-CSCC). IEEE; 2024. p. 1–6.

[37] Zhang F, Zhan J, Wang Y, Cheng J, Wang M, Chen P, et al. Enhancing thalassemia gene carrier identification in non-anemic populations using artificial intelligence erythrocyte morphology analysis and machine learning. Eur J Haematol. 2024;112(5):692–700. doi: 10.1111/ejh.14160 38154920

[38] Vaishnavi K, Nikhitha Kamath U, Ashwath Rao B, Subba Reddy NV. Predicting mental health illness using machine learning algorithms. J Phys: Conf Ser. 2022;2161(1):012021. doi: 10.1088/1742-6596/2161/1/012021

[39] Saleem M, Aslam W, Lali MIU, Rauf HT, Nasr EA. Predicting Thalassemia using feature selection techniques: A comparative analysis. diagnostics. 2023;13(22):3441. doi: 10.3390/diagnostics13223441 37998577 PMC10670018

[40] Nadimpalli RR, Jalapally M, Acereda A. Feature engineering for mental health applications. Wearable AI in psychotherapy. IGI Global Scientific Publishing; 2026. p. 153–82.

[41] Kaushik P, Jain E, Gill KS, Upadhyay D, Devliyal S. Optimizing mental health prediction by fine-tuning decision classifier parameters for enhanced accuracy. In: 2024 2nd international conference on sustainable computing and smart systems (ICSCSS); 2024. p. 935–9. 10.1109/icscss60660.2024.10625480

[42] Azijah N, Ratna S, Muflih M, Budiman H, Syapotro U, Ariyani K. Classification of mental health care using the ELM, MLP, and CatBoost stacking framework. J Data Sci. 2024;2024.

[43] Ko J, Oh S, Enkhbayar D, Lee J, Chung M-K, Shin T, et al. Interpretable feature selection and hybrid deep learning models for major depressive disorder prediction from wearable device data. Springer Science and Business Media LLC; 2025. 10.21203/rs.3.rs-6747824/v1 PMC12956937 41774238

[44] Magboo VPC, Magboo MSA. Important features associated with depression prediction and explainable ai. In: International Conference on Well-Being in the Information Society; 2022. p. 23–36.

[45] Bashar N, Ahmed MdS, Haque Mili M, Mary MM. A hybrid clustering and classification approach for mental health diagnosis in Rohingya refugees using explainable AI: SHAP and LIME. In: 2025 2nd international conference on next-generation computing, IoT and machine learning (NCIM); 2025. p. 1–6. 10.1109/ncim65934.2025.11159903

[46] Pendyala V, Kim H. Assessing the reliability of machine learning models applied to the mental health domain using explainable AI. Electronics. 2024;13(6):1025. doi: 10.3390/electronics13061025

[47] Hameed S, Nauman M, Akhtar N, Fayyaz MA, Nawaz R. Explainable AI for mental health: Detecting mental illness from social media using NLP and machine learning. Front Artif Intell. 2025;8:1627078. 41018736 10.3389/frai.2025.1627078 PMC12460309

[48] Mazzuca D, Bergantin F, Macrì D, Zinno F, Forestiero A. AI approach for enhanced thalassemia diagnosis using blood smear images. In: pHealth 2024. IOS Press; 2024. p. 123–4. 10.3233/SHTI240072 38785016

[49] Phanthong B, Charoenkwan P, Kamlungkuea T, Luewan S, Tongsong T. Accuracy of red blood cell parameters in predicting *α*0-Thalassemia trait among non-anemic males. J Clin Med. 2025;14(10):3591. doi: 10.3390/jcm14103591 40429585 PMC12111872

[50] Hossain MJ, Islam MW, Munni UR, Gulshan R, Mukta SA, Miah MS, et al. Health-related quality of life among thalassemia patients in Bangladesh using the SF-36 questionnaire. Sci Rep. 2023;13(1):7734. doi: 10.1038/s41598-023-34205-9 37173392 PMC10182078

[51] Ahsan M, Mahmud M, Saha P, Gupta K, Siddique Z. Effect of data scaling methods on machine learning algorithms and model performance. Technologies. 2021;9(3):52. doi: 10.3390/technologies9030052

[52] Prasetiyowati MI, Maulidevi NU, Surendro K. Determining threshold value on information gain feature selection to increase speed and prediction accuracy of random forest. J Big Data. 2021;8(1). doi: 10.1186/s40537-021-00472-4

[53] Liu Y, Li Y, Xie D. Implications of imbalanced datasets for empirical ROC-AUC estimation in binary classification tasks. J Stat Comput Simul. 2023;94(1):183–203. doi: 10.1080/00949655.2023.2238235

[54] Dheenathayalan K, Savitha K. Mental disorder classification using ensemble machine learning. In: International conference on mathematical modeling and computational science; 2025. p. 149–59.

[55] Amirruddin AD, Muharam FM, Ismail MH, Tan NP, Ismail MF. Synthetic minority over-sampling TEchnique (SMOTE) and logistic model tree (LMT)-adaptive boosting algorithms for classifying imbalanced datasets of nutrient and chlorophyll sufficiency levels of oil palm (Elaeis guineensis) using spectroradiometers and unmanned aerial vehicles. Comput Electron Agric. 2022;193:106646. doi: 10.1016/j.compag.2021.106646

[56] Ali S, Abuhmed T, El-Sappagh S, Muhammad K, Alonso-Moral JM, Confalonieri R, et al. Explainable artificial intelligence (XAI): What we know and what is left to attain Trustworthy Artificial Intelligence. Inform Fusion. 2023;99:101805. doi: 10.1016/j.inffus.2023.101805

[57] Zafar MR, Khan N. Deterministic local interpretable model-agnostic explanations for stable explainability. MAKE. 2021;3(3):525–41. doi: 10.3390/make3030027

[58] Sihi D, Dari B, Kuruvila AP, Jha G, Basu K. Explainable machine learning approach quantified the long-term 1981 –2015) impact of climate and soil properties on yields of major agricultural crops across CONUS. Front Sustain Food Syst. 2022;6. doi: 10.3389/fsufs.2022.847892

[59] Siddique Ayon S, Ebrahim Hossain H, Ullah Miah MS, Rahman MM, Mahmud M. Explainable AI in feature selection: Improving classification performance on imbalanced datasets. In: International conference on neural information processing; 2024. p. 303–18.

[60] Google Research. Colaboratory. https://colab.research.google.com/

[61] James V. Miranda L. PySwarms: A research toolkit for Particle Swarm Optimization in Python. JOSS. 2018;3(21):433. doi: 10.21105/joss.00433

[62] Fernández V, Amor13 ˘053’fn R, Firpo V, Morisset C. LIME: A line measuring library for large and complex spectroscopic data sets-I. Implementation of a virtual observatory for JWST spectra. Astron Astrophys. 2024;688:A69.

[63] scikit-learn developers. Scikit-learn: Machine learning in python; 2025.

[64] Nti IK, Nyarko-Boateng O, Aning J. Performance of machine learning algorithms with different K values in K-fold CrossValidation. IJITCS. 2021;13(6):61–71. doi: 10.5815/ijitcs.2021.06.05

